# A non-aggressive, highly efficient, enzymatic method for dissociation of human brain-tumors and brain-tissues to viable single-cells

**DOI:** 10.1186/s12868-016-0262-y

**Published:** 2016-06-01

**Authors:** Ilan Volovitz, Netanel Shapira, Haim Ezer, Aviv Gafni, Merav Lustgarten, Tal Alter, Idan Ben-Horin, Ori Barzilai, Tal Shahar, Andrew Kanner, Itzhak Fried, Igor Veshchev, Rachel Grossman, Zvi Ram

**Affiliations:** Cancer Immunotherapy Laboratory, Department of Neurosurgery, Tel Aviv Sourasky Medical Center, Weizmann 6, Tel Aviv, Israel; Department of Neurosurgery, Tel Aviv Sourasky Medical Center, Weizmann 6, Tel Aviv, Israel; Department of Neurosurgery, Galilee Medical Center, Lohamei HaGeta’ot 5, Nahariya, Israel

**Keywords:** Brain tumors, Glioma, Glioblastoma, Brain metastasis, Brain, Tissue dissociation, Neutral protease, Dispase, Collagenase, DNase

## Abstract

**Background:**

Conducting research on the molecular biology, immunology, and physiology of brain tumors (BTs) and primary brain tissues requires the use of viably dissociated single cells. Inadequate methods for tissue dissociation generate considerable loss in the quantity of single cells produced and in the produced cells’ viability. Improper dissociation may also demote the quality of data attained in functional and molecular assays due to the presence of large quantities cellular debris containing immune-activatory danger associated molecular patterns, and due to the increased quantities of degraded proteins and RNA.

**Results:**

Over 40 resected BTs and non-tumorous brain tissue samples were dissociated into single cells by mechanical dissociation or by mechanical and enzymatic dissociation. The quality of dissociation was compared for all frequently used dissociation enzymes (collagenase, DNase, hyaluronidase, papain, dispase) and for neutral protease (NP) from *Clostridium histolyticum*. Single-cell-dissociated cell mixtures were evaluated for cellular *viability* and for the cell-mixture *dissociation quality*. *Dissociation quality* was graded by the quantity of subcellular debris, non-dissociated cell clumps, and DNA released from dead cells. Of all enzymes or enzyme combinations examined, NP (an enzyme previously not evaluated on brain tissues) produced dissociated cell mixtures with the highest mean cellular *viability*: 93 % in gliomas, 85 % in brain metastases, and 89 % in non-tumorous brain tissue. NP also produced cell mixtures with significantly less cellular debris than other enzymes tested. Dissociation using NP was non-aggressive over time—no changes in cell viability or dissociation *quality* were found when comparing 2-h dissociation at 37 °C to overnight dissociation at ambient temperature.

**Conclusions:**

The use of NP allows for the most effective dissociation of viable single cells from human BTs or brain tissue. Its non-aggressive dissociative capacity may enable ambient-temperature shipping of tumor pieces in multi-center clinical trials, meanwhile being dissociated. As clinical grade NP is commercially available it can be easily integrated into cell-therapy clinical trials in neuro-oncology. The high quality viable cells produced may enable investigators to conduct more consistent research by avoiding the experimental artifacts associated with the presence dead cells or cellular debris.

**Electronic supplementary material:**

The online version of this article (doi:10.1186/s12868-016-0262-y) contains supplementary material, which is available to authorized users.

## Background

Investigating the physiology, molecular biology and immunology of brain BTs [1] frequently requires the use of viable single cells produced by dissociation of tumor pieces collected from patients undergoing craniotomy. Several methods are used to dissociate the tumor mass into viable single cells. These include mechanical dissociation (e.g. meshing, trituration with a pipette/tip) [2–5], enzymatic digestion [4, 6–11], or a combination of both. Enzymes such as papain [6, 7], dispase [6, 8, 9], collagenase [4, 6, 8–11], hyaluronidase [4, 11], DNase [4, 9–11], and trypsin [12, 13] are commonly used for dissociation, either alone or in combination. Enzymes dissociate the cell–cell contacts and the extracellular matrix (ECM) encompassing cells within the brain tissue or inside the BT [14].

The various dissociation methods largely differ in their yield of cells [15, 16] and in the percentage of viable cells produced [17]. The produced cell mixtures (i.e. the cells and their surrounding solution) may differ in their dissociation quality i.e. the undissociated cell clumps, the extent of subcellular debris, and the amount of spilt nucleic acids [17].

Inefficient or overly aggressive tumor dissociation may cause the release of cellular materials that constitute DAMPs or alarmins [18]. Such materials include glutamate [19], ATP [20], HMGB1 [21] and others [22]. The released cellular components may activate, modulate or selectively kill the assayed cells thereby producing significant experimental artifacts [2, 15, 16, 23]. Inappropriate tissue dissociation may also compromise the quality of functional assays that require intact viable cells. It may reduce the accuracy of the results of molecular assays such as gene expression assays that require genetic material of suitable integrity [13], and may alter the results of flow cytometry (FCM) that correctly analyze only intact single cells [17, 24].

In addition to their use in research, brain tumor cells dissociated from surgical specimen are used in clinical trials for production of whole-cell vaccines [25]. Vaccination with live, dead or dying cells results in different immunological responses [26, 27]. In preparation for a clinical trial using viable dissociated glioblastoma cells as vaccines [26], we sought an optimal dissociation method that could produce single cells of the highest possible viability and of the optimal dissociation quality using enzymes approved for clinical use.

To evaluate which enzyme or enzyme combination produces single cells of the highest dissociation quality from dissociated brain lesions, all commonly used enzymes were tested on a large set of non-tumorous brain lesions and BT samples. Our results show that NP from *Clostridium histolyticum*, an enzyme not previously used on human brain lesions, produced single cells of the highest viability and cell mixtures of the finest dissociation quality. NP’s non-aggressive nature enabled long term incubations with no apparent reduction in the dissociated cells’ viability or in the dissociation quality.

## Methods

### Human subjects

BT tissue samples were obtained from patients aged 25–81 years who underwent surgical procedures at the Neurosurgery Department at Tel-Aviv Medical Center. BTs were pathologically classified by neuropathologists. Brain tissue samples were obtained from three patients harboring BTs during the surgical approach to deep seated tumors and from three epileptic patients whose epileptic foci were removed.

### Brain tissue dissociation to single cells

Freshly isolated brain tissue and BT tissue was transported to the lab in saline or in Ringer lactate (Biological Industries, Beit HaEmek, Israel). The specimens were weighed following the removal of blood clots and necrotic areas. The cleansed tissue was cut into 1–2 mm pieces and resuspended in HBSS^(+Ca+Mg)^ without phenol red (Biological Industries) at 100 mg tissue per ml. The tumor slurry was divided into 4 ml aliquots per 50 ml tube to allow for complete trituration using a 5 ml plastic Pasteur pipette (Biologix, Zouqu, China).

The following enzymes or their combination were tested on the tumor slurry:

1. DNase-I (Sigma St. Louis, MO, USA, Cat.—AMP-D1): an endonuclease used to reduce viscosity (‘gooeyness’) resulting from DNA released from dead cells [11, 28, 29]. Optimal concentration—5 units/ml (u/ml).
2. Collagenase type IV from *Clostridium histolyticum* (Sigma, Cat.—M9070): a metalloprotease that cleaves native triple-helical collagen [11, 29, 30] found in ECM. Optimal concentration—0.05 %.
3. Papain from papaya latex (Sigma, Cat.—p3125): a relatively nonspecific protease [29, 31].
4. Hyaluronidase type V from sheep testis (Sigma, Cat.—H6254): an enzyme hydrolyzing glycosidic linkages in hyaluronic acid found in ECM. It is typically used as a supplement when performing dissociation with other enzymes [11, 29, 32]. Optimal concentration—1000 u/ml.
5. Dispase-II from *Bacillus polymyxa* (Sigma Cat.—D4693): a non-specific metalloprotease that cleaves fibronectin and collagen IV + I, but not collagen V or laminin. It hydrolyzes peptide bonds of non-polar amino acid residues [9, 29]. Optimal concentration—0.6 u/ml.
6. Neutral protease (NP) from *Clostridium histolyticum* (AMSBio-Abingdon, UK, Cat.—30301): a metalloprotease that hydrolyzes peptide bonds of non-polar amino acid residues. The enzyme is free from collagenolytic activity [29, 33]. Optimal concentration—0.11 DMC u/ml.

Different enzymes were added to the slurry-containing tubes, tubes were swirled and left with unlocked caps either in room temperature (RT) overnight (ON), or incubated for 30′, 60′, or 120′ at 37 °C. Following incubation, the tumor tissue was triturated 5–8 times using a 5 ml plastic Pasteur pipette, which was pressed towards the bottom of the tube. Triturated tumor cells were then briefly swirled and after approximately 30 s, large undigested debris that settled at the bottom of the tube was collected and discarded. The cell mixtures were then washed twice with PBS^−Ca–Mg^ (Biological Industries) at 400 rcf and a sample from the cell mixture was stained with trypan blue (Sigma) and microscopically evaluated.

### Evaluating cellular viability using the trypan-blue exclusion method and Red blood cell exclusion

The standard trypan blue dye-exclusion method was used to evaluate cellular viability.

Red blood cells (RBC), which were frequently a significant portion of the cells produced, were removed by ACK RBC lysis buffer (Lonza, Allendale, NJ, USA) according to the manufacturer’s protocol. Alternatively RBC were not removed, but microscopically identified and disregarded while counting. Dissociated tumor, brain and immune cells have variable shapes and sizes that can be occasionally mistaken for RBC. RBC can be identified as the smallest, round, trypan blue excluding cells within the dissociated cell mixture.

### Evaluating the dissociation quality of tissue dissociation

After evaluating for cellular viability, the cell mixture was inspected for the dissociation quality. A simple grading system for cell-mixture *dissociation quality* was devised by evaluating three main parameters of dissociation quality—cell clumps, subcellular debris and DNA debris. In order to reduce evaluation subjectivity, each parameter was evaluated on a 1–3 scale, where 1 represents much debris, 2—little debris and 3—no debris. A cumulative grade (CG) for the quality of dissociation is given as the sum of the three dissociation parameter grades. The CG ranges from 3 to 9, where a CG of 9 indicates a clean cell-mixture containing only single cells (live or dead) without any debris.

The evaluated dissociation quality parameters were:

1. *Cell clumps*—Conglomerates of cells that did not dissociate into single cells.
2. *Subcellular debris/remnants*—Fragments which are irregular in shape and smaller than any of the dissociated cells.
3. “Gooeyness”—DNA spilt from dead cells. DNA debris are much larger than any cell, and appear as long semi-translucent strands in which many cells are entwined.

### Freezing and thawing dissociated cells

Dissociated tumor/brain cells were frozen in fetal calf serum (FCS) (HyClone, Cramlington, UK) + 10 % DMSO (Sigma) [34]. Controlled rate cooling was achieved using isopropanol-filled “Mr Frosty” (Thermo Scientific, Nalgene, Rochester, NY, USA). The cells were kept in a −80 °C until evaluation.

Cells were thawed at 37 °C and collected from their freezing ampoule using a 10× volume of pre-warmed medium with serum (DMEM [Biological Industries], 10 % FCS and combined antibiotics) or using a defined serum-free medium (X-VIVO™-15, Lonza). Following thawing, cells were left untouched in medium at 37 °C for at least 1–2 h before evaluating their viability/dissociation-quality or using them for any downstream assays [34, 35].

### Flow cytometric evaluation of the cells’ viability

Dissociated cells were stained with ViViD (violet viability dye)—an amine reactive fixable viability dye (Molecular Probes, Invitrogen, Eugene, OR, USA) according to manufacturer’s protocol. The cells were washed in PBS^−/−^ and fixed by adding 250 µl of 1 % formaldehyde (Electron Microscopy Sciences, Hatfield, PA, USA) in PBS^−/−^. Cells were acquired using the Canto-II flow cytometer (BD biosciences). The data files were analyzed using Flow-Jo (Tree Star, Ashland, OR, USA).

### Statistical evaluation

Student’s independent samples two-tailed t test was used for statistical comparison of dissociation quality. Results are expressed as means with standard error (SE) unless stated otherwise. P-value was considered significant where P < 0.05. N represents the number of biological samples tested.

## Results

### Comparison of tumor dissociation quality by dispase, papain, or a combination of DNase, collagenase and/or hyaluronidase

The first set of six, side-by-side, experiments was conducted solely on glial tumors. Enzymes evaluated were DNase [4, 9–11], collagenase with [4, 11] or without [10] hyaluronidase, papain [6, 7] and dispase [8, 9]. Trypsin was not tested as it was reported to generate significant loss of viable cells and membranal antigen cleaving [6, 17]. Mechanical dissociation was used in this set of experiments as a control for enzymatic digestion.

The enzyme concentration-ranges tested were obtained from the product data sheets or from published literature using the selected enzymes. The following concentration ranges were evaluated: papain (2–20 u/ml), dispase (0.6–2.4 u/ml), DNase (1–20 u/ml), collagenase (0.02–0.2 % W/V), and hyaluronidase (200–4000 u/ml). High, medium and low concentrations of each enzyme were evaluated for their dissociative ability during 30, 60, or 120 min incubations or during ON incubation. All combinations of DNase, collagenase, with or without hyaluronidase, at different concentrations, were also tested.

**Fig. 1:**
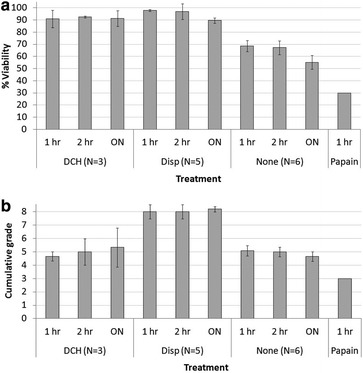
Brain tumor (BT) dissociation to single cells using various enzymes. **a** Cellular viability and **b** dissociation cumulative grade (CG) for BTs dissociated with dispase (Disp), papain, a combination of DNase, collagenase and hyaluronidase (DCH), or mechanical dissociation only (none). See text for calculation of CG. Primary brain tumors were dissociated to single cells for 1 hour (1 h), 2 hour (2 h), or overnight (ON) at optimal enzyme concentrations—(see text). After the indicated times, the cells were triturated using a Pasteur pipette and their viability and CG was determined. *Statistics:* Viability of Disp or DCH dissociated-tumors to mechanically dissociated tumors (P < 0.0005 or less). CG of Disp-1 h to none-1 h and to DCH-1 h (P < 0.0001 either). CG of Disp-2 h to None-2 h and DCH-2 h (P < 0.025 either). CG of dispase ON to none-ON (P < 0.0001)

 Figure 1a, b depicts only the optimal enzyme concentrations for each enzyme/combination that were determined for a dissociation durations of 1, 2 h and ON (a 30 min incubation gave markedly inferior results). Optimal enzyme concentrations determined were: DNase (5 u/ml), collagenase (0.05 %) and hyaluronidase (1000 u/ml). The dissociation with DNase and collagenase without hyaluronidase is not shown, as dissociation with DNase + collagenase + hyaluronidase (DCH) produced superior dissociation quality and viability at comparable concentrations.

Figure 1a depicts the percentage of viable cells following tissue dissociation. Cellular viability was the highest following dissociation with dispase. DCH tested in three experiments produced comparable high viability. Enzyme unassisted mechanical dissociation by trituration of the tumor slurry produced significantly lower viabilities (P < 0.0005), and was discontinued after six experiments. Papain was discontinued after one experiment since it produced inferior results even in comparison to mechanical dissociation, yielding very low numbers of viable cells.

Figure 1b shows the quality of dissociation—graded using the CG scoring. Unlike the comparable viability produced by dispase versus DCH, dispase-dissociated tumors produced cell mixtures of significantly higher quality than those dissociated with DCH or using mechanical dissociation. Again, tumors dissociated with papain attained a CG that is lower than those that were mechanically dissociated.

Taken together, the initial set of experiments indicate that although DCH and dispase yielded mixtures with comparable viabilities, the cell mixtures qualities produced were significantly higher for dispase (P < 0.0001). We therefore continued to the next set of experiments with dispase only.

### Comparison of tumor dissociation with dispase versus NP for short durations

Following dissociation of a total of 15 brain tumors and brain metastases using dispase (a neutral protease from *Bacillus polymyxa*), we searched for a supplier offering clinical-grade dispase that may be used to produce viable whole cells for vaccination of glioma patients. As no clinical grade dispase was found, we tested another neutral protease from a different microorganism—NP from *Clostridium histolyticum* (NP), an enzyme offered by several companies both in clinical-grade and in non-clinical grade.

**Fig. 2:**
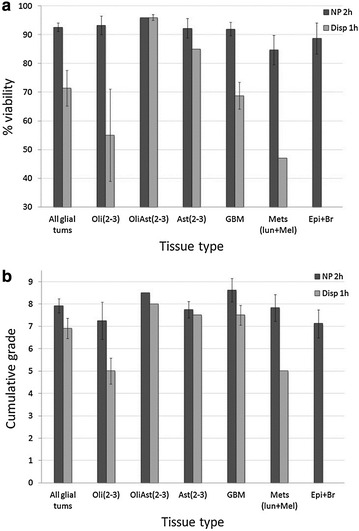
BT dissociation to single cells using dispase (Disp) or neutral protease (NP). **a** Cellular viability and **b** dissociation quality (CG) of BTs dissociated with dispase or NP, at the respective enzyme’s optimal dissociation time. Following indicated times (1 or 2 h) the cells were triturated using a Pasteur pipette and their viability and CG were determined. Oli—Oligodenderoglioma, OliAst–Oligoastrocytoma, Ast–Astrocytoma, GBM–Glioblastoma, Mets–Metastasis to the brain (lung–lun and melanoma–Mel), Epi + Br—Epileptic foci, and peritumoral brain tissue. *Parenthesis* indicate the grade of the tumors, e.g. Oli(2–3). *Statistics* Viability following dissociation of all glial tumors (Oli, OliAst, Ast, GBM) using NP-2 h to dispase-1 h (P < 0.01)

Figure 2a, b compares tissue dissociation with dispase versus NP. For brevity, only the optimal time durations for dissociation using the two enzymes were compared (1 h for dispase, and 2 h for NP at 37 °C). Interestingly, although dispase and NP are both neutral proteases (hydrolyzing peptide bonds of non-polar amino acid [29, 33]), they displayed considerable differences in quality of dissociation. Breite et al. [33] compared these two enzymes in acellular in vitro assays and showed that they differed in their proteolytic activities.

Figure 2a shows that NP yielded consistently higher viabilities in the produced cell mixtures compared to dispase, for all types of tissues tested. Combining all dissociated glial tumors (11× dispase vs. 15× NP), NP yielded significantly higher viability mixtures than dispase (P < 0.01), with a mean of >90 % cellular viability of dissociated glial tumors.

Dissociation with NP also showed consistently better quality of cell mixtures (Fig. 2b). Although no significant differences were found between the CG scores of NP versus dispase, the evaluation of CG’s parameters (clumps, remnants and gooeyness) revealed that short-term dissociation with NP produced less cell clumps, and significantly less subcellular-debris (remnants) than dispase (P < 0.03). Both enzymes produced cell mixtures that were usually devoid of DNA debris (Additional file 1: Figure S1).

### Comparison of tumor dissociation quality between dispase and NP overnight

Labs may receive tissue from the operating room at late hours. The development of a protocol for cell dissociation for longer dissociation durations may allow to initiate tissue dissociation while receiving the tissue (e.g. in the afternoon) and conclude it the next morning. Ambient temperature dissociation for extended durations may also facilitate dry-ice-free inexpensive air freight of tissues/tumors. The tissues, harvested on one site, will be slowly dissociated, meanwhile being transferred to a central site/lab.

**Fig. 3:**
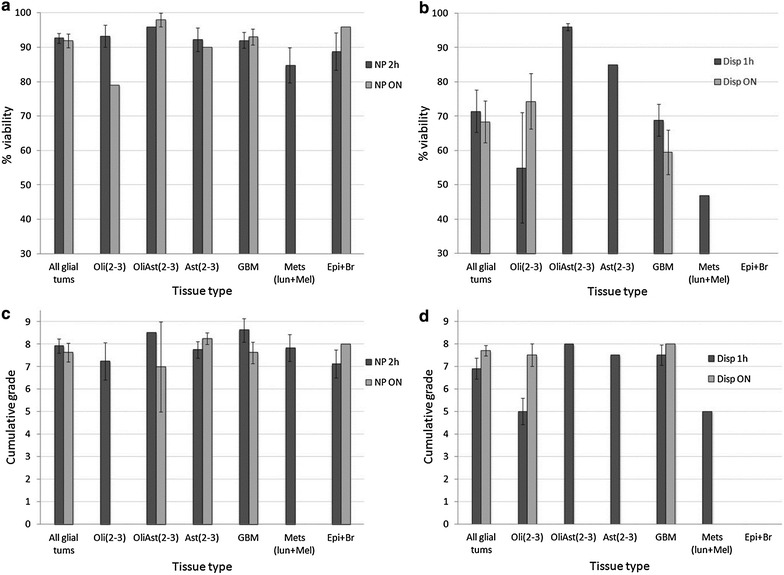
BT dissociation ON to single cells using dispase or NP. **a** Cellular viability and **b** dissociation quality of NP-2 h versus NP-ON. **c** Cellular viability and **d** dissociation quality of dispase-1 h versus dispase-ON. BTs were dissociated for 1–2 h or ON. Following indicated times, the cells were triturated and their viability and CG was determined. *Statistics* Viability of dissociated cells and the dissociation quality was not different for all glial tumors between NP 2 h to NP-ON, or between dispase 1 h to dispase-ON

Figure 3a–d shows the viability and the dissociation quality of BTs and brain tissues dissociated overnight (ON) either with dispase or with NP at room temperature. The figures show that NP or dispase produced similar quality mixtures comparing shorter (1–2 h) versus longer (ON) durations (Additional file 1: Figure S1). In contrast, ON dissociation with NP produced cell mixtures of higher viability and better dissociation quality than dispase.

Other ON dissociation methods such as dissociation at 37 °C, or keeping minced-but-undissociated tumor at 4 °C ON then dissociating the tumor at 37 °C for 1–2 h, both yielded inferior cellular viabilities, and dissociation qualities than ON dissociation at ambient temperature (not shown).

### Viable cell outputs following tissue dissociation using dispase or NP

Cell yields following dissociation of GBM tissue by NP or dispase samples was compared. As different tumors harbor different numbers of cells, dispase and NP were compared only for GBM tissue, in which there were sufficient number of samples to enable comparison. Similar viable cell yield per gram of GBM tissue were produced by dispase (6.2 ± 4.1 × 10^7^ cells (N = 6)) and by NP (7.6 ± 4.3 × 10^7^ cells (N = 9)) (P = 0.54).

Table 1 summarizes the viable cell yields from all dissociations of glial tumors, brain metastases and brain tissue samples using dispase and NP, a total of 47 dissociations. The table combines data from the dispase and the NP-dissociated tissues, having similar cell yields, to attain larger sample sizes and thereby more accurate cell-yield estimates. The high natural variability in cell yields of BTs can be appreciated by the large ranges of cells attained per gram of tissue even in tumors of the same grade. The average viable cell output per gram of anaplastic astrocytomas (grade III) was 1.35 × 10^8^ while GBMs (grade IV astrocytomas) yielded about half these numbers (7.3 × 10^7^ cells/g). Melanomas and lung brain-metastases yielded 6.4 × 10^7^ cells/g, and non-tumoral brain tissue yielded 1.15 × 10^8^ cells/g in epileptic foci to 2.89 × 10^8^ cells/g in peritumoral brain. The differences between the two types of non-tumoral brain tissue is likely due to the different brain areas from which samples were obtained, but may also be due to small sample size of these rare tissue specimen.

**Table 1:** Viable cell yields of dissociated brain tumors and brain tissue

Tissue type (grouped)	Tissue subtype	N = NP/Disp	Mean × 10^6^ cells/g	STD × 10^6^	Range × 10^6^ cells/g
All glial tumors	All primary glial tumors	21/15	102	70	19–376
*Oli (2*–*3)*	Oli-2	5/6	107	106	19–376
	Oli-3		81	44	30–112
*OliAst* (*2*–*3*)	OliAst-2	1/2	96	1	96–97
	OliAst-3		87	–	87
*Ast* (*2*–*3*)	Ast-2	6/1	111	18	100–132
	AA		135	72	37–189
*GBM*	GBM	9/6	70	42	21–136
Mets	Melanoma + lung	4/1	64	35	34–117
Epil + brain	Epileptic focus	5/0	115	7	110–120
	Brain		289	330	99–670

### Freezing and thawing of dissociated brain/tumor cells

**Fig. 4:**
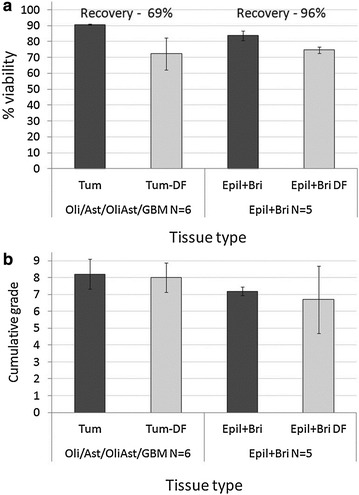
Cellular viability and CG of freshly dissociated cells versus thawed cells. BTs or brain tissue were dissociated using NP and graded for viability, recovery (**a**) and for dissociation quality (**b**), immediately after NP dissociation or following freezing and thawing (defrosting—DF). Cellular viability and CG were determined using trypan blue

A significant decline in the number of cells recovered following freezing and thawing is a known phenomenon for brain cells [34, 36]. Figures 4a, b follow the fate of brain and BT cells dissociated by NP, frozen, and thawed using standard freezing procedures [34]. Figure 4a shows that following thawing, the fraction of viable BT cells decreased from 91 to 72 %; the cell recovery rate (i.e. the number of live cells recovered divided by those frozen) was 69 %. The fraction of viable brain cells decreased from 84 to 75 %, with cell-recovery of 96 %. These recovery rates are higher than those previously reported for human (55–60 % [34]) or for rat (56 %) brain cells [36].

Figure 4b follows the dissociation quality of the brain tissue or the BT cell mixtures before freezing and after thawing, showing no significant changes. In our experience, cell mixtures that have low dissociation quality before freezing are usually associated with lower yields of recovered cells after thawing.

DNA debris significantly reduces the cell yields after thawing, thus in mixtures of lower dissociation quality, the addition of DNA-hydrolyzing enzymes like DNase or Benzonase to the thawing medium is warranted.

### Monitoring the cellular viability using a FCM viability dye and trypan-blue exclusion method

Viability of dissociated cells can either be evaluated microscopically using dye exclusion, or flow-cytometrically using a viability dye [35, 37]. Viability dyes that distinguish between live and dead cells are frequently integrated into antibody staining panels; antibodies nonspecifically bind to dead cells and can generate major FCM artifacts [24, 38]. Here, a fixable amine viability dye (ViViD) that stains amine groups was used to determine cellular viability [24, 38].

**Fig. 5:**
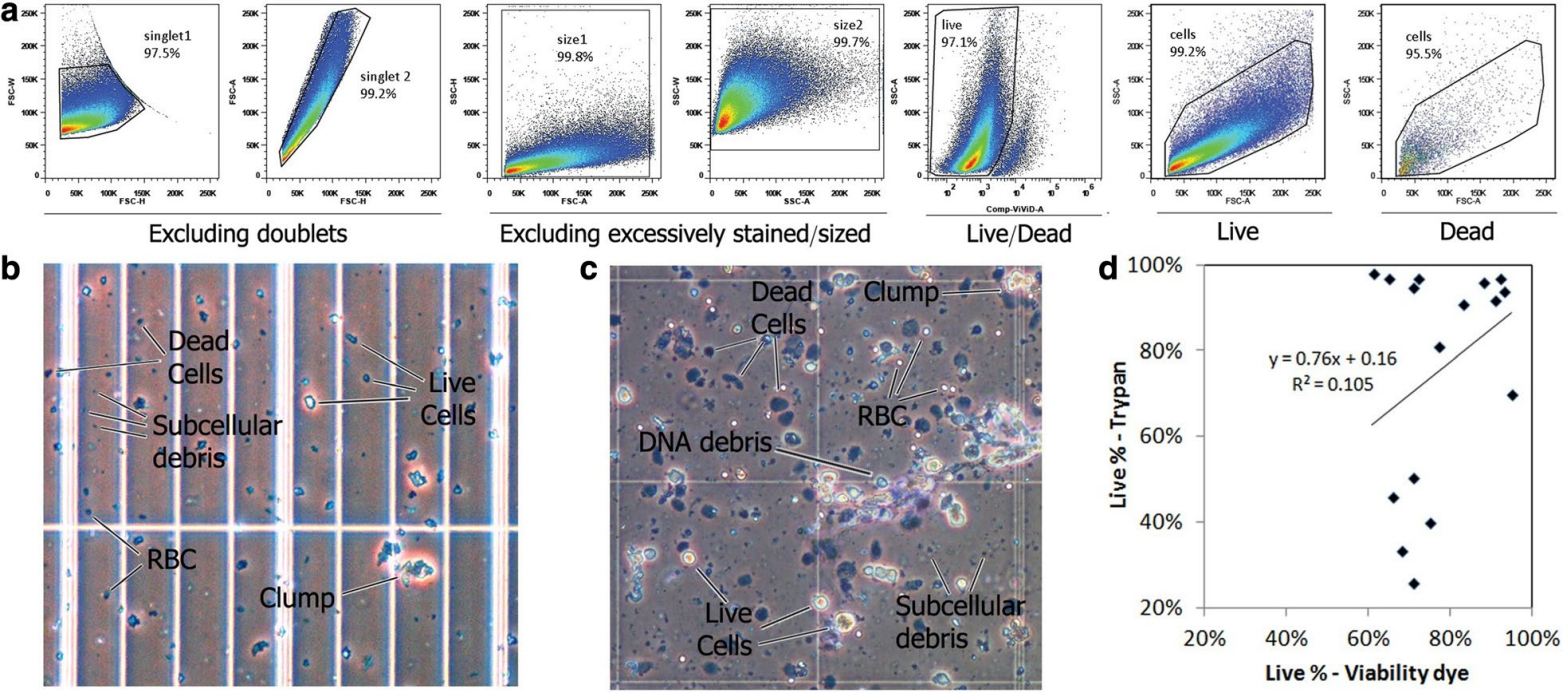
Comparison between trypan-blue and flow-cytometry to determine cellular viability. **a** Cells dissociated from a GBM sample, stained with ViViD, an amine-reactive viability dye, and flow cytometrically analyzed (**b**) photographs of dissociated (**c**) brain cells and (**d**) BT cells, stained using trypan blue. **d** Correlation between percent viability determined by trypan blue (mean = 77 %) and flow cytometry (mean = 75 %), sixteen samples depicted

Figure 5a depicts a dissociated GBM sample serially gated for viability. The first two gates remove doublet and clumped cells, gating-in only singlet cells (sin) [35]. The next two gates remove excessively stained or sized cells laying on the far axes; these cells introduce artifacts into the flow cytometric analysis [35, 38]. The next dot-plot discriminates between dead (ViViD^high^) and live (ViViD^low^) cells. The last two dot plots illustrate that it is impossible to discriminate between live and dead human tumor (or brain) cells based solely on their FSC/SSC plots, a method previously used in FCM to determine viability.

Figure 5b, c depicts microscopic evaluation of viability and dissociation quality. The mixtures were mechanically dissociated brain (5b) and BT samples (5c) of low dissociation quality selected to illustrate as many visually-identifiable dissociation quality issues. Figure 5b, which depicts trypan-blue stained dissociated brain tissue, exhibits the following objects: *Live cells*—usually irregular in shape, with a shiny body, and a light halo around them. *Dead cells*—irregular in shape, with a darker body. *RBC*—round cells, smaller than all other cells. *Sub*-*cellular debris*—very small, usually dark. *Clumps*—several cells clustered together.

Figure 5c depicts trypan-blue stained high grade glioma exhibiting the following objects: live cells, dead cells, sub-cellular debris, RBC, and *DNA debris*—semi-translucent strands in which cells are entwined. The visual discrimination between live and dead cells is generally more difficult with brain tissue than with BTs. Parallel trypan-blue staining of blood-borne leukocytes helps in the identification and quantification of viable cells.

Figure 5d compares the percent viable cells for 16 samples that were evaluated for viability in parallel by trypan-blue and by FCM. The samples consist of 12 BTs and 4 brain tissue samples. The mean viability determined by trypan was 77, and 75 % by FCM (P = NS). While at high viabilities the percent of viable cells evaluated by either method was similar, in other cases the methods gave somewhat dissimilar viability counts—without any consistency for one method to overestimate viability.

## Discussion

Here we investigated all widely used methods and enzymes employed for dissociation of BTs and brain tissue using the largest panel of tissue samples used for such comparison. Unlike previous work, we added a visual grading system, CG, for the evaluation of the dissociation quality component of the produced cell mixtures.

Cell mixtures of lower dissociation quality generally yielded fewer cells. More importantly, cell mixtures of higher dissociation quality contained less components released from dead or dying cells. Cellular debris contains DAMPs, substances to which brain-resident immune cells, and brain-infiltrating immune cells respond. The presence of DAMPs in large quantity may alter the results of functional experiments using the dissociated cells [2, 15, 16, 18–22].

While DCH, the most widely used method to produce single cells from human BTs, generated single cells of similar viability as that of dispase, it produced mixtures of significantly lower dissociation quality. Although dispase produced cell mixtures of acceptable viability and dissociation quality, there is no commercially available clinical-grade version of this enzyme. In contrast, NP is an inexpensive enzyme which is available in clinical and non-clinical grades. Importantly, NP was found to dissociate brain tissues significantly better than dispase, both in regard to cellular viability and to dissociation quality.

In addition to NP’s ability to gently dissociate brain/tumor tissue for short duration (2 h), it dissociated tissues for longer durations at ambient temperature without any apparent reduction in the produced cellular viability or the dissociation quality.

Neutral proteases are not inhibited by serum and can be used in cell culture media [39] to inhibit formation of cell clumps. Thus it may be possible to transport brain tissues or BTs at ambient temperatures in tissue-culture medium with or without serum, supplemented with NP. The tissue obtained from patients at one clinical site could be sent in culture medium with NP, and processed as *fresh tissue* at a distant site. This may facilitate multi-center collaborations requiring centralized processing of fresh tissue samples.

NP is not of eukaryotic origin, thus carries no risk of spongiform encephalopathy. Its clinical-grade version is made under GMP guidelines, and was previously used in trials in which the dissociated cells, e.g. pancreatic islet cells [40] were returned to humans. This enables the simple integration of this enzyme into clinical trials in the field of neuroscience.

Figure 5d demonstrated some discrepancy between cellular viabilities evaluated by trypan-blue and by FCM. This previously reported discrepancy [41–43] is likely due to the fact that microscopy and FCM identify differently what is “a cell”. Microscopy identifies cells via their shape; while blood cells are microscopically easily identifiable, brain or BT cells are highly irregular (see Fig. 5b, c) and sometimes difficult to identify. FCM, on the other hand, identifies cells by their light scatter characteristics. “Cells” are electronically collected “events” above a somewhat arbitrary forward scatter threshold. Also in FCM, cellular identification is complicated by the irregularity of the cells and the high variability in their sizes. Another complicating factor for FCM is that the dissociated cell mixtures may contain large amounts of cellular debris. While the use of an amine dye does a good job at discriminating between live and dead cells, it is less efficient in discriminating between live cells and debris, both having low fluorescence in the viability dye channel.

Which method should be used to evaluate viability? Microscopy may be better at correctly identifying cells and more widely accepted by regulatory agencies. On the other hand FCM is rapid, quantitative and more user-independent thus enabling standardization of analysis and comparison of viabilities across different samples dissociated by different labs [42, 43].

Using the high dissociation quality cell mixtures produced from BTs and brain samples enables our lab to run elaborate multicolor (up to 10 colors) FCM analyses and FCM sorting experiments of intratumoral cells. When using the dissociated BT cells in functional immune assays (e.g. co-culturing of tumor cells with lymphocytes) we see that cell mixtures of low dissociation quality yield atypical results.

Calibration of an optimal way to dissociate brain tissues or BTs into viable cells is important both clinically and scientifically. *Clinically*, intact BT cells used for immunotherapy trial should contain minimal amounts of debris, and maximal amounts of viable cells, whether cells are viable cells [26] or irradiated [25, 44]. *Scientifically*, the production of better quality cell mixtures is the first important step for attaining more consistent and reliable results in the field of neuroscience.

## Conclusions

Neutral protease (NP) from *Clostridium histolyticum,* an enzyme previously not used in the field of neuroscience, dissociates human brain tissue and brain tumors to single cells with significantly higher viabilities and cleaner cell-mixtures than all other widely-used enzymes. The non-aggressive nature of NP allows for tissue dissociation for extended durations, enabling for ambient-temperature shipping of fresh tissue pieces meanwhile being dissociated.

Improper tissue dissociation may reduce the quality of data attained in functional and molecular assays due to the presence of large numbers of necrotic cells, spilt nucleic acids, and the presence of subcellular debris, containing immune-activatory danger associated molecular patterns (DAMPs). Production of high-quality viable single cells from brain tissue is the first step for more consistent and reliable results in the field.

## Additional file

10.1186/s12868-016-0262-y Grading of dissociation quality of all glial tumors. The three different parameters accounting for the dissociation cumulative grade-CG, i.e. Clumps, Remnants and Gooeyness, were graded following tumor dissociation using NP -2hr, dispase- 1hr, NP-ON and dispase-ON. The parameters were graded from 1 to 3, with 1 representing low dissociation quality and 3- high dissociation quality culture (see materials and methods). *Statistics:* Cell remnants following dissociation using NP-2hr to dispase-1hr (P < 0.03).

## Abbreviations

BTs: brain tumors
CG: cumulative grade
DCH: DNase + collagenase + hyaluronidase
FCM: flow cytometry
NP: neutral protease
ON: overnight
RBC: red blood cells
RT: room temperature
ViViD: violet viability dye

## References

[1] do Carmo A, Balca-Silva J, Matias D, Lopes MC (2013). PKC signaling in glioblastoma. Cancer Biol Ther.

[2] Hayashida Y, Partida GJ, Ishida AT (2004). Dissociation of retinal ganglion cells without enzymes. J Neurosci Methods.

[3] Nagato M, Heike T, Kato T, Yamanaka Y, Yoshimoto M, Shimazaki T, Okano H, Nakahata T (2005). Prospective characterization of neural stem cells by flow cytometry analysis using a combination of surface markers. J Neurosci Res.

[4] Gomez GG, Kruse CA (2004). Isolation and culture of human brain tumor cells. Methods Mol Med.

[5] Louis SA, Mak CK, Reynolds BA (2012). Methods to culture, differentiate, and characterize neural stem cells from the adult and embryonic mouse central nervous system. Methods Mol Biol.

[6] Panchision DM, Chen HL, Pistollato F, Papini D, Ni HT, Hawley TS (2007). Optimized flow cytometric analysis of central nervous system tissue reveals novel functional relationships among cells expressing CD133, CD15, and CD24. Stem Cells.

[7] Patel AP, Tirosh I, Trombetta JJ, Shalek AK, Gillespie SM, Wakimoto H, Cahill DP, Nahed BV, Curry WT, Martuza RL (2014). Single-cell RNA-seq highlights intratumoral heterogeneity in primary glioblastoma. Science.

[8] Yan Y, Xu Y, Gao Y-Y, Zong Z-H, Zhang Q, Li C, Wang HQ (2013). Implication of 14-3-3ε and 14-3-3θ/τ in proteasome inhibition-induced apoptosis of glioma cells. Cancer Sci.

[9] Pistollato F, Persano L, Puppa AD, Rampazzo E, Basso G (2011). Isolation and expansion of regionally defined human glioblastoma cells in vitro. Curr Protoc Stem Cell Biol.

[10] Rebetz J, Tian D, Persson A, Widegren B, Salford LG, Englund E, Gisselsson D, Fan X (2008). Glial progenitor-like phenotype in low-grade glioma and enhanced CD133-expression and neuronal lineage differentiation potential in high-grade glioma. PLoS One.

[11] Sawamura Y, Abe H, Aida T, Hosokawa M, Kobayashi H (1988). Isolation and in vitro growth of glioma-infiltrating lymphocytes, and an analysis of their surface phenotypes. J Neurosurg.

[12] Singh SK, Hawkins C, Clarke ID, Squire JA, Bayani J, Hide T, Henkelman RM, Cusimano MD, Dirks PB (2004). Identification of human brain tumour initiating cells. Nature.

[13] Vrtacnik P, Kos S, Bustin SA, Marc J, Ostanek B (2014). Influence of trypsinization and alternative procedures for cell preparation before RNA extraction on RNA integrity. Anal Biochem.

[14] Ziu M, Schmidt NO, Cargioli TG, Aboody KS, Black PM, Carroll RS (2006). Glioma-produced extracellular matrix influences brain tumor tropism of human neural stem cells. J Neurooncol.

[15] Wolters GH, Vos-Scheperkeuter GH, Lin H-C, van Schilfgaarde R (1995). Different roles of class I and class II *Clostridium histolyticum* collagenase in rat pancreatic islet isolation. Diabetes.

[16] Van Deijnen J, Van Suylichem P, Wolters G, Van Schilfgaarde R (1994). Distribution of collagens type I, type III and type V in the pancreas of rat, dog, pig and man. Cell Tissue Res.

[17] Maric D, Barker JL (2004). Neural stem cells redefined. Mol Neurobiol.

[18] Kox M, Pompe JC, Pickkers P, Hoedemaekers CW, van Vugt AB, van der Hoeven JG (2008). Increased vagal tone accounts for the observed immune paralysis in patients with traumatic brain injury. Neurology.

[19] Choi DW (1992). Excitotoxic cell death. J Neurobiol.

[20] Tsuda M, Beggs S, Salter MW, Inoue K (2013). Microglia and intractable chronic pain. Glia.

[21] Pisetsky DS, Erlandsson-Harris H, Andersson U (2008). High-mobility group box protein 1 (HMGB1): an alarmin mediating the pathogenesis of rheumatic disease. Arthritis Res Ther.

[22] Palucka K, Banchereau J (2012). Cancer immunotherapy via dendritic cells. Nat Rev Cancer.

[23] Yamazoe H, Iwata H (2006). Efficient generation of dopaminergic neurons from mouse embryonic stem cells enclosed in hollow fibers. Biomaterials.

[24] Perfetto SP, Chattopadhyay PK, Lamoreaux L, Nguyen R, Ambrozak D, Koup RA, Roederer M (2006). Amine reactive dyes: an effective tool to discriminate live and dead cells in polychromatic flow cytometry. J Immunol Methods.

[25] Mitchell DA, Fecci PE, Sampson JH (2008). Immunotherapy of malignant brain tumors. Immunol Rev.

[26] Volovitz I, Marmor Y, Azulay M, Machlenkin A, Goldberger O, Mor F, Slavin S, Ram Z, Cohen IR, Eisenbach L (2011). Split immunity: immune inhibition of rat gliomas by subcutaneous exposure to unmodified live tumor cells. J Immunol.

[27] Shurin MR, Potapovich AI, Tyurina YY, Tourkova IL, Shurin GV, Kagan VE (2009). Recognition of live phosphatidylserine-labeled tumor cells by dendritic cells: a novel approach to immunotherapy of skin cancer. Cancer Res.

[28] Davidson DJ, Gray MA, Kilanowski FM, Tarran R, Randell SH, Sheppard DN, Argent BE, Dorin JR (2004). Murine epithelial cells: isolation and culture. J Cyst Fibros.

[29] Worthington K, Worthington V. Worthington enzyme manual. Worthington Biochemical Corporation; 2011. http://www.worthington-biochem.com/pap/default.html.

[30] Seglen PO (1976). Preparation of isolated rat liver cells. Methods Cell Biol.

[31] He W, Ingraham C, Rising L, Goderie S, Temple S (2001). Multipotent stem cells from the mouse basal forebrain contribute GABAergic neurons and oligodendrocytes to the cerebral cortex during embryogenesis. J Neurosci.

[32] Hwang WS, Roh SI, Lee BC, Kang SK, Kwon DK, Kim S, Kim SJ, Park SW, Kwon HS, Lee CK (2005). Patient-specific embryonic stem cells derived from human SCNT blastocysts. Science.

[33] Breite AG, Dwulet FE, McCarthy RC (2010). Tissue dissociation enzyme neutral protease assessment. Transplant Proc.

[34] Silani V, Pizzuti A, Strada O, Falini A, Buscaglia M, Scarlato G (1988). Human neuronal cell viability demonstrated in culture after cryopreservation. Brain Res.

[35] Lamoreaux L, Roederer M, Koup R (2006). Intracellular cytokine optimization and standard operating procedure. Nat Protoc.

[36] Higgins AZ, Cullen DK, LaPlaca MC, Karlsson JO (2011). Effects of freezing profile parameters on the survival of cryopreserved rat embryonic neural cells. J Neurosci Methods.

[37] Betts MR, Nason MC, West SM, De Rosa SC, Migueles SA, Abraham J, Lederman MM, Benito JM, Goepfert PA, Connors M (2006). HIV nonprogressors preferentially maintain highly functional HIV-specific CD8+ T cells. Blood.

[38] Perfetto SP, Chattopadhyay PK, Roederer M (2004). Seventeen-colour flow cytometry: unravelling the immune system. Nat Rev Immunol.

[39] Mena I, Roussarie JP, Brahic M (2004). Infection of macrophage primary cultures by persistent and nonpersistent strains of Theiler’s virus: role of capsid and noncapsid viral determinants. J Virol.

[40] Szot GL, Lee MR, Tavakol MM, Lang J, Dekovic F, Kerlan RK, Stock PG, Posselt AM (2009). Successful clinical islet isolation using a GMP-manufactured collagenase and neutral protease. Transplantation.

[41] Hilpert F, Heiser A, Wieckhorst W, Arnold N, Kabelitz D, Jonat W, Pfisterer J (2005). The impact of electrical charge on the viability and physiology of dendritic cells. Scand J Immunol.

[42] Wigg AJ, Phillips JW, Wheatland L, Berry MN (2003). Assessment of cell concentration and viability of isolated hepatocytes using flow cytometry. Anal Biochem.

[43] Humpe A, Beck C, Schoch R, Kneba M, Horst HA (2005). Establishment and optimization of a flow cytometric method for evaluation of viability of CD34+ cells after cryopreservation and comparison with trypan blue exclusion staining. Transfusion.

[44] Okada H, Kohanbash G, Zhu X, Kastenhuber ER, Hoji A, Ueda R, Fujita M (2009). Immunotherapeutic approaches for glioma. Crit Rev Immunol.

