# Supplementary figures and images for: A non-aggressive, highly efficient, enzymatic method for dissociation of human brain-tumors and brain-tissues to viable single-cells
Source: BMC Neurosci. 2016 Jun 1;17:30. doi: 10.1186/s12868-016-0262-y (PMC4888249; doi:10.1186/s12868-016-0262-y)

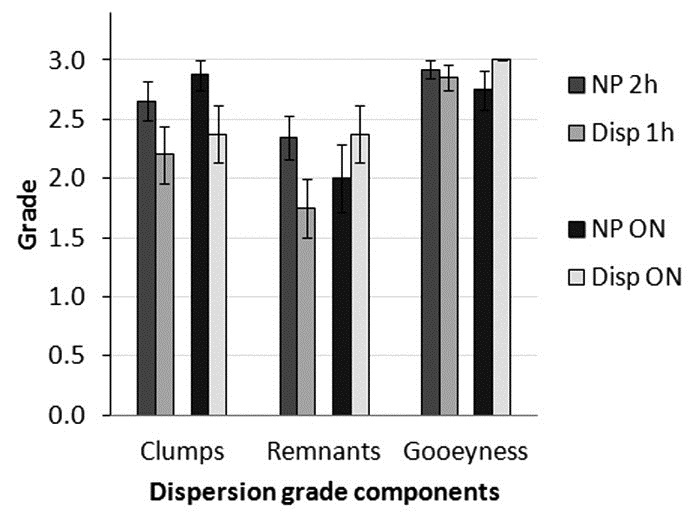

Supplement: Supplementary file 1 — 10.1186/s12868-016-0262-y Grading of dissociation quality of all glial tumors. The three different parameters accounting for the dissociation cumulative grade-CG, i.e. Clumps, Remnants and Gooeyness, were graded following tumor dissociation using NP -2hr, dispase- 1hr, NP-ON and dispase-ON. The parameters were graded from 1 to 3, with 1 representing low dissociation quality and 3- high dissociation quality culture (see materials and methods). Statistics: Cell remnants following dissociation using NP-2hr to dispase-1hr (P < 0.03). [file 12868_2016_262_MOESM1_ESM.jpg]
